# Synthetic and Semisynthetic Compounds as Antibacterials Targeting Virulence Traits in Resistant Strains: A Narrative Updated Review

**DOI:** 10.3390/antibiotics12060963

**Published:** 2023-05-25

**Authors:** Dejan Stojković, Jovana Petrović, Tamara Carević, Marina Soković, Konstantinos Liaras

**Affiliations:** 1 Department of Plant Physiology, Institute for Biological Research “Siniša Stanković”—National Institute of the Republic of Serbia, University of Belgrade, Bulevar Despota Stefana 142, 11000 Belgrade, Serbia; dejanbio@ibiss.bg.ac.rs (D.S.); jovana0303@ibiss.bg.ac.rs (J.P.); tamara.carevic@ibiss.bg.ac.rs (T.C.); mris@ibiss.bg.ac.rs (M.S.); 2 Department of Life and Health Sciences, School of Sciences and Engineering, University of Nicosia, 2417 Nicosia, Cyprus

**Keywords:** antibiotic resistance, virulence factors, novel synthetic compounds, biofilms, antibacterial activity

## Abstract

This narrative review paper provides an up-to-date overview of the potential of novel synthetic and semisynthetic compounds as antibacterials that target virulence traits in resistant strains. The review focused on research conducted in the last five years and investigated a range of compounds including azoles, indoles, thiophenes, glycopeptides, pleuromutilin derivatives, lactone derivatives, and chalcones. The emergence and spread of antibiotic-resistant bacterial strains is a growing public health concern, and new approaches are urgently needed to combat this threat. One promising approach is to target virulence factors, which are essential for bacterial survival and pathogenesis, but not for bacterial growth. By targeting virulence factors, it may be possible to reduce the severity of bacterial infections without promoting the development of resistance. We discuss the mechanisms of action of the various compounds investigated and their potential as antibacterials. The review highlights the potential of targeting virulence factors as a promising strategy to combat antibiotic resistance and suggests that further research is needed to identify new compounds and optimize their efficacy. The findings of this review suggest that novel synthetic and semisynthetic compounds that target virulence factors have great potential as antibacterials in the fight against antibiotic resistance.

## 1. Introduction

Antibiotic resistance is a major global health challenge, threatening the efficacy of currently available antibiotics [1]. The emergence and spread of multidrug-resistant bacteria underscore the urgent need for new antibacterial agents that can overcome resistance mechanisms [2]. Estimates indicate that, annually, over 2 million infections caused by resistants strains occur worldwide, with as many as approximately 30,000 fatal outcomes in the USA alone and USD 5 billion in health care assets allocated to this issue. At the beginning of the 21st century, a list of pathogenic microorganisms that showed different levels of resistance to antimicrobial agents was released, and it included *Enterococcus faecium*, *Staphylococcus aureus*, *Klebsiella pneumoniae*, *Acinetobacter baumannii*, *Pseudomonas aeruginosa*, and *Enterobacter* spp. The list is also known as the famous register of ESKAPE pathogens. Traditional antibiotics target bacterial growth, which can lead to the development of resistance through the acquisition of mutations or the transfer of resistance genes [3]. In contrast, compounds that target bacterial virulence traits, such as biofilm formation, quorum sensing, and motility, may be less prone to the development of resistance [4]. Bacterial infections remain a significant challenge in public health, and the emergence of antimicrobial resistance (AMR) has further complicated the treatment of bacterial infections [5]. Despite the availability of many antibiotics, the prevalence of bacterial infections caused by multidrug-resistant bacteria has increased alarmingly. One strategy to overcome AMR is to target the virulence traits of bacterial pathogens, which are distinct from traditional antibiotic targets [6]. Virulence factors are the attributes that enable pathogens to cause disease in a host, such as adhesion, invasion, colonization, and the secretion of toxins and enzymes [7]. Therefore, the inhibition of virulence factors is a promising approach to combating bacterial infections [8].

The virulence traits of resistant bacteria have received increasing attention in recent years. Biofilm formation is one of the important virulence factors that contribute to bacterial resistance [9]. Biofilms are complex communities of microorganisms that are encased in a self-produced extracellular matrix, which confers resistance to antibiotics and immune defense mechanisms [10]. Bacteria can cause disease by producing agents known as virulence traits, which are specific compounds produced by bacteria that allow them to evade the host’s immune system response. Virulence traits such as quorum sensing, motility, and iron acquisition have also been reported to be involved in the pathogenicity and antibiotic resistance of bacterial pathogens. As well as adhesins, invasins, and antiphagocytic factors, toxins, hemolysins, and proteases are among the agents that cause harm to the host [11].

**Figure 1:**
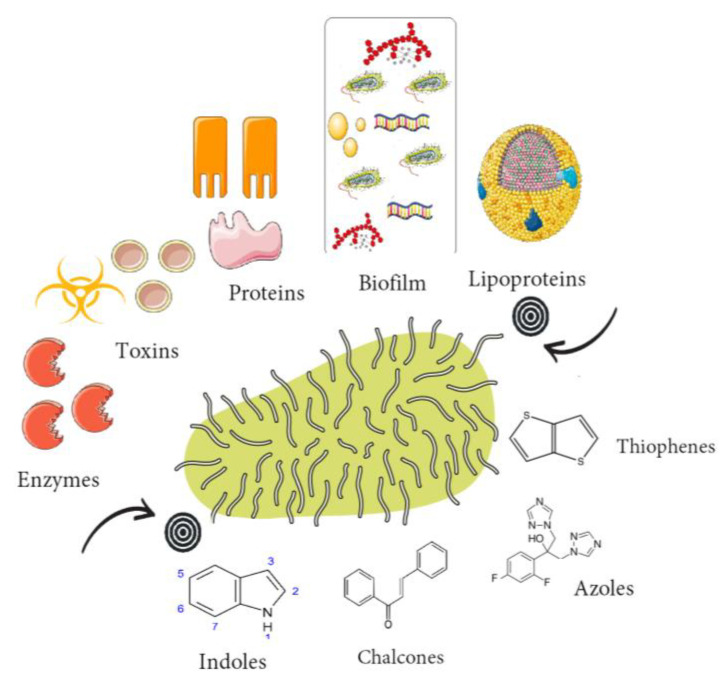
Group of compounds that target virulence traits in resistant bacteria.

A variety of natural and synthetic compounds have been reported to possess anti-virulence activity against resistant bacteria. Among them, azoles, indoles, thiophenes, glycopeptides, pleuromutilin derivatives, lactone derivatives, and chalcones have been found to exhibit promising antivirulence activity [12,13,14,15]. These compounds target various virulence factors and interfere with the pathogenicity of bacterial pathogens (Figure 1), thus enhancing the efficacy of antibiotics and reducing the emergence of resistance.

Chalcones are a class of natural and synthetic compounds that have been shown to inhibit bacterial biofilm formation and quorum sensing [16]. Azoles, including pyrazoles, oxadiazoles, and triazoles, have been extensively studied as antifungal agents, but recent studies have shown that they also have antibacterial activity against resistant strains [17]. Coumarins have been found to inhibit bacterial quorum sensing and motility [18], while indoles and thiophenes have also shown potential as quorum sensing inhibitors [19]. Quinolines have been proposed as inhibitors of bacterial type II topoisomerases and have shown activity against multidrug-resistant bacteria [20]. Terpenoids, including triterpenoids and other scaffolds, have been mainly studied as semisynthetic analogues with promising antibacterial activity [21]. Glycopeptides, such as vancomycin and teicoplanin, have been widely used as antibiotics, but semisynthetic analogues have been developed to overcome resistance mechanisms [22]. Pleuromutilin derivatives, including retapamulin and lefamulin, have been approved for clinical use and have shown efficacy against resistant strains [23]. Finally, albocyclin and other lactone derivatives have shown activity against Gram-positive and Gram-negative bacteria, including resistant strains [24].

Targeting virulence traits is an attractive strategy to combat resistant bacteria. The use of compounds that target virulence factors can complement traditional antibiotic therapies, leading to enhanced efficacy and reduced resistance. Therefore, continuous research on the development of anti-virulence compounds and their mechanisms of action is crucial in the fight against AMR. In this review, we discuss recent studies on synthetic and semisynthetic compounds with antibacterial activity, focusing on their ability to target virulence traits in resistant strains. We also highlight the potential of these compounds to overcome antibiotic resistance mechanisms and suggest directions for future research. The presented review article mainly summarizes the progress in this field in the last 5 years, but its also covers some older important data. The fresh perspectives of compounds newly identified as potential therapeutics targeting virulence factors are presented, along with the established antimicrobial properties of certain novel compounds and the repurposing of existing antibacterial/antifungal therapeutics.

## 2. Indoles

Indoles, a widespread naturally occurring class of alkaloid compounds, are not only important bacterial intercellular signal molecules, but also a crucial component of the amino acid tryptophan. They are a particularly intriguing class of compounds covering a range of pharmacological activities, including antiinflammatory, antihistaminic, antitumor, antioxidative, and antidiabetic properties. Research covering this topic is quite important due to the versatile nature of indole compounds, which may lead to numerous chemical modifications, i.e., presenting possibilities for drug development. With respect to the subject of this review, their antibacterial potential, particularly their targeting of virulence factors, is thoroughly elaborated herein. 

**Figure 2:**
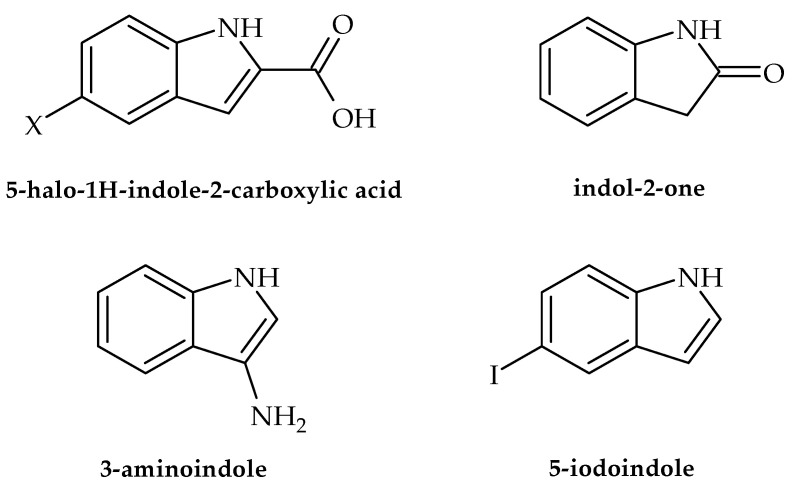
Chemical structures of 5-halo-*1H*-indole-2-carboxylic acid, indol-2-one, 3-amino indole, and 5-iodoindole.

In the following section, we present the antibacterial potential of selected indole-derived compounds against clinically relevant strains, namely the causative agents of urinary and skin infections as well as gastroenteritis-causing bacteria. According to Balcerek et al. [25], commercial compounds, such as 5-halo-*1H*-indole-2-carboxylic acids, (Figure 2) were efficient against a panel of bacterial strains, particularly *Listeria monocytogenes*. The obtained results indicated that this activity may be used for the development of medicines in the treatment of listeriosis in cases when resistance/allergy is present. Along with these results, assays also showed that indol-2-one (Figure 2) with a morpholinosulfonyl component acted as a potent inhibitor of the DNA gyrase of both Gram-positive and Gram-negative bacteria, with activity against *S. aureus* even better than ciprofloxacin (IC_50_ values 18.75 µM and 26.43 µM, respectively) [26]. Furthermore, Alzahrani et al. [27] showed that novel derivatives of the compound thiazolo-indolin-2-one exerted rather promising antibacterial activity, with a noteworthy ability to affect virulence traits such as biofilm formation in *S. aureus* (ATCC 29213) and *P. aeruginosa* (biofilm inhibition concentration (BIC_50_) of 1.95 µg/mL and 3.9 µg/mL, respectively). As for the ability to affect traits of *A. baumanii*, literature data indicate that d-pyrimido[4,5-*b*] indole derivatives show inhibitory potential against this pathogen in the range of 0.25–1 gmL^−1^ [28]. Furthermore, 3-amino indoles (Figure 2), 4-hydroxy-2-pyridone derivatives containing indolyl, 2-hydrazino2-imidazoline, and bis-indolyl methane Schiff bases have also been identified as potential antimicrobial agents that may also inhibit the growth of MDR *A. baumanii*. Recent research conducted by Raorane et al. [29] showed that halogenated indole 5-iodoindole (Figure 2) promptly affected the development and motility of *A. baumannii*, disrupted its biofilm formation, and eventually eradicated this pathogenic microorganism as effectively as ciprofloxacin and gentamicin. This was achieved via the development of ROS, which had a profound influence on the integrity of the plasma membrane, eventually leading to a loss of bacterial viability. Furthermore, the tested compound turned out to be verry effective against *Escherichia coli* and *S. aureus* but did not influence the viability of *P. aeruginosa*. According to Kim et al. [30], indole and its derivatives also proved efficient in the inhibiting single-species and multi-species biofilms of the acne-forming bacterial skin strains *Cutibacterium acnes* and *S. aureus*, with 3,3′-diindolylmethane as the most potent inhibitor. The obtained results indicated that indole-derived compounds may be useful in developing efficient skin treatments related to the tested bacteria.

**Figure 3:**
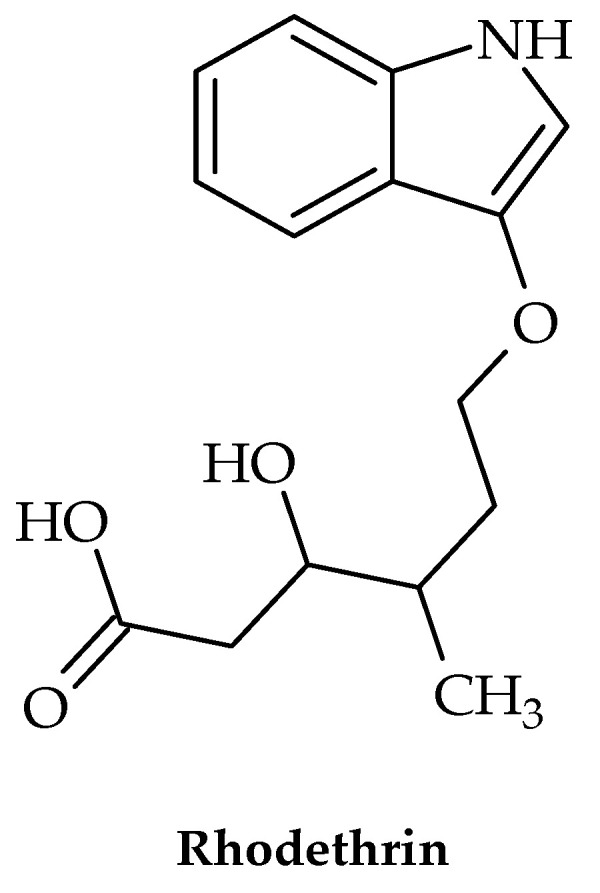
Chemical structure of rhodethrin.

The eradication of the nosocomial pathogen *Enterococcus faecalis* has been shown to be quite challenging in recent years, with biofilm development and resistance to antibiotics as the two main causes. Hence, new treatments are urgently needed, particularly those affecting these two traits. A study by Tatta et al. [31] showed that the indole terpenoid compound rhodethrin (Figure 3) in combination with chloramphenicol disrupted the overall formation of biofilm, which may lead to the easier and more effective treatment of vancomycin-resistant *E. faecalis*.

Nosocomial urinary infections related to catheter application are most often caused by *Proteus mirabilis*. Due to biofilm development, they have been increasingly harder to treat, leading to a demand for novel and efficient treatments. Hence, Amer et al. [32] developed new Foley catheters impregnated with indole compounds (indole extract from the supernatant of the rhizobacterium *Enterobacter* sp. Zch127) in order to disrupt the biofilm formation of *Proteus mirabilis*. The results showed a reduction in the formation of biofilm of 60–70% in terms of biomass, which was confirmed by the expression of virulence genes responsible for biofilm formation, while genes that regulate the formation of capsular polysaccharides were not affected. The catheters were considered safe for use, since they had no cytotoxic effects on fibroblasts. Along with nosocomial *P. mirabilis*, uropathogenic *E. coli* is a common inhabitant of the human urinary tract, leading to recurrent infections. The recurrence rate depends on the pathogen’s ability to infiltrate the urinary epithelium and evade host defense mechanisms. Boya et al. [33] demonstrated that 4-chloroindole, 5-chloroindole, and 5-chloro 2-methyl indole may profoundly impact biofilm formation at an average dose of 20 g/mL by as much as 67%, along with their ability to reduce bacterial motility, necessary for colony dispersal. A more in-depth study showed that the tested compounds affected the expression of genes related to adhesion and toxin production, which may be of importance in managing clinical manifestations of these health conditions.

Due to their ability to regulate internal environments by removing toxic substances, efflux pumps are an important target when considering the development of new drugs. According to Cernicchi et al. [34], indole derivatives could also have wide-ranging applications in this area, which could increase their use in clinical practice.

Along with the fact that indoles are highly active against pathogenic microorganisms of clinical relevance, they have also been shown to be very efficient in targeting virulence traits of *Agrobacterium tumefaciens*. This may be rather important with respect to the economy, since this microorganism is known as a plant pathogen causing significant lossess in various crops. As Ahmed et al. [35] demonstrated in their study, among 83 indole derivatives that were tested against *A. tumefaciens*, 4-chloroindole, 6-iodoindole, and 5-chloro-2-methyl indole inhibited its growth at doses as high as 50 μg mL^−1^. Furthermore, they also affected virulence factors such as swimming motility, the production of exopolysaccharide and exoprotease, and cell surface hydrophobicity and biofilm formation. 

Besides issues with various crops, the aquaculture sector also faces a serious problem resulting from bacterial infections. In terms of money, losses resulting from vibriosis—a disease caused by *Vibrio campbelli*—are quite substantial. This has inevitably led to the development of novel and sustainable strategies required for managing problems in the aquaculture industry. One of these is the evaluation of indole analogs’ activity against *V. campbellii*, probably the main bacterial pathogens in aquaculture. Out of 44 tested compounds, 17 halogenated indoles (including 6-bromoindole, 7-bromoindole, 4-fluoroindole, 5-iodoindole, and 7-iodoindole) have been shown to affect the virulence traits of *V. campbellii.* Furthermore, they have been found to increase the survival of brine shrimp, used as a valid in vivo system model, by over 80% at 10 mM, as well as to affect virulence traits such as swimming motility and biofilm formation (at concentrations of 10 mM and 100 mM), whereas only mild inhibition was achieved with the tested concentrations regarding protease activity. The absence of hemolytic activity was observed using the tested concentrations [36]. Similar antibacterial virulence-targeting activity was previously obtained for *Vibrio tasmaniensis* LGP32 and *Vibrio crassostreae* J2-9, used as two model infections of bivalves [36], which indicates that this strategy may be very useful in developing antivirulence therapy. The control of *Vibrio parahaemolyticus*, a potential cause of gastroenteritis brought on by the consummation of raw sea food, is also becoming increasingly important, since a certain amount of healthcare expenses have been directed towards treating this condition. In their study, Sathiyamoorthi et al. [37] demonstrated that halogenated indole derivatives (4-chloroindole, 7-chloroindole, 4-iodoindole, and 7-iodoindole) strongly influence some of the virulence factors of *V. parahaemolyticus*: for example, 4-chloroindole inhibited biofilm formation by 80% at a MIC of 50 g/mL, whereas 100 g/mL terminated its viability within the first 30 min of activity. As it turned out, the position of the halogenated substituent in indole core determines its extraordinary activity. 

Though these results did not highlight the potential of indole compounds to target bacterial virulence factors, recent data published by Li et al. [38] showed that 5-methylindole instantly eradicated several bacterial strains, including *S. aureus*, *E.faecalis*, *E. coli*, *P. aeruginosa*, methicillin-resistant *S. aureus*, *K. pneumoniae*, and *Mycobacterium tuberculosis*.

## 3. Azoles

Azole derivatives are heterocyclic compounds comprised of a nitrogen atom and at least one other non-carbon atom (such as nitrogen, sulfur, or oxygen) as part of the ring. They encompass a wide number of derivatives, such as thiadiazole, oxadiazole, triazole, imidazole, isoxazole, and pyrazole. Mainly known as antifungal agents, azole derivatives demonstrate many other biological properties, including antidiabetic, immunosuppressant, antiinflammatory, and anticancer activities. Even though they were initially used for the treatment of fungal infections, various azole-containing compounds have been shown to inhibit the growth of bacteria as well, via a different mode of action. In fungi, azoles mainly inhibit the production of ergosterol—an essential component of the fungal plasma membrane—whereas in bacteria, their activity is based on the fact that the attachment of azole to bacterial flavohemoglobin (protein) eventually leads to the increasing production of ROS, which have fatal effects on bacterial viability [39].

Due to their versatility in chemical structure and biological activities, azoles have been widely investigated in pharmacochemistry, but they still present surprises. According to Srikanth et al. [28], azole compounds are highly efficient against *A. Baumanii,* which is of great importance considering that this multi-drug-resistant pathogenic microorganism belongs to the infamous ESKAPE group. In particular, naphthalimide-containing nitroimidazoles with decyl-piperazine exerted strong activity against *A. baumannii* (MIC 0.013 MmL^−1^) and, combined with norfloxacin, eradicated even the resistant strains. Additionaly, ammonium containing imidazoles also showed antimicrobial potential. The same study demonstrated that the type of the modification as well as the substituent determines the level of antimicrobial properties. Thus, the presence of 4-Br-phenol modification increased activity against *A. baumanii*, whereas a hydrophobic *n*-butyl chain on the phenyl ring decreased activity against the same pathogen. The absence of a halogen molecule is generally reflected through a decrease in bioactivity.

**Figure 4:**
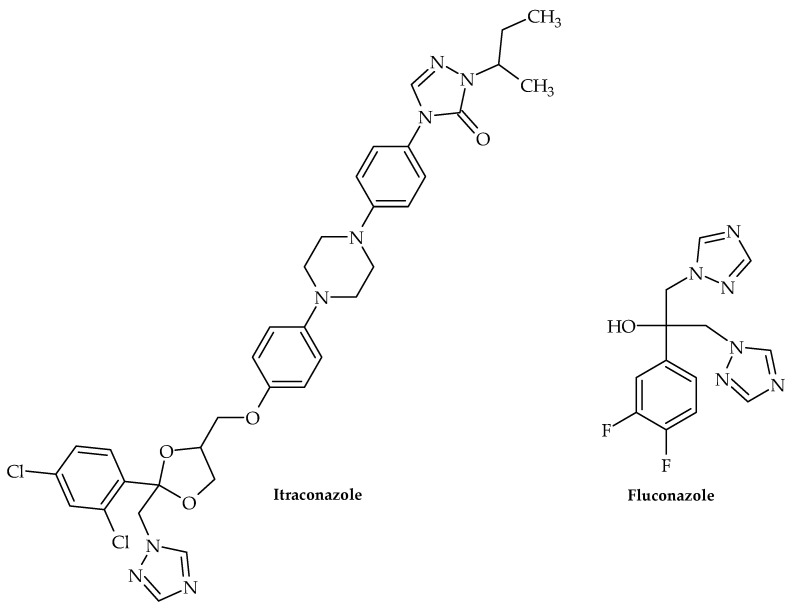
Chemical structures of itraconazole and fluconazole.

**Figure 5:**
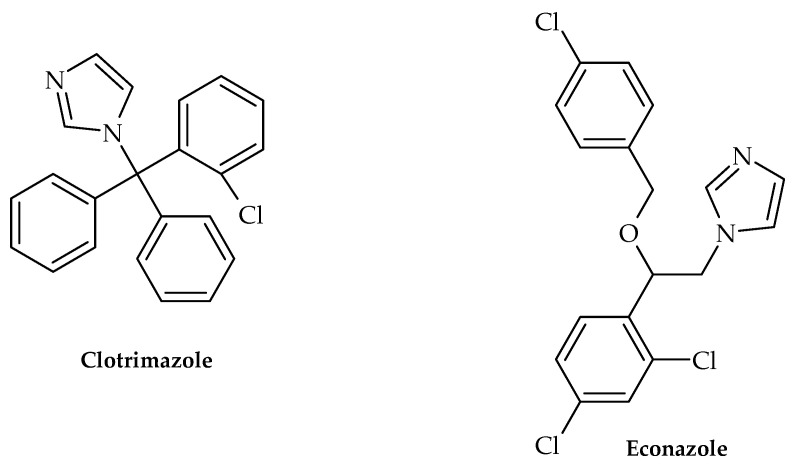
Chemical structures of clotrimazole and econazole.

Along with this growing trend of repurposing already available therapeutics, Olaifa et al. [40] also investigated the ability of itraconazole and fluconazole (Figure 4) to target specific virulence factors. The ability to disrupt biofilm formation in *A. baumanii* was demonstrated in the abovementioned study, which clearly indicated that azole compounds may very well be underinvestigated in terms of their antibacterial and virulence-targeting potential. This was also previously demonstrated by Qiu et al. [41]—using *Streptococcus mutans* clinical isolates as model organisms, clotrimazole and econazole (Figure 5) inhibited its growth at 12.5 and 25 mgL^−1^, respectively. Furthermore, they were able to inhibit biofilm production, which undoubtedly demonstrated that these antifungal medicines may also target bacterial virulence factors.

**Figure 6:**
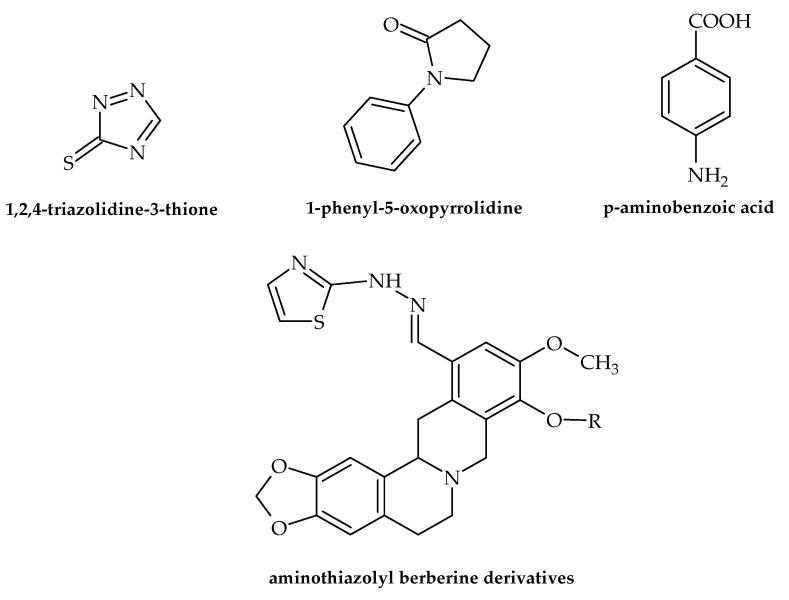
Chemical structures of 1,2,4-triazolidine-3-thione, 1-phenyl-5-oxopyrrolidine, p-aminobenzoic acid, and aminothiazolyl berberine.

Numerous data dealing with the antibacterial potential of antifungal drugs have been presented in the last two years. Even though these drugs do not target virulence factors, the results are noteworthy, favoring the repurposing of antifungal drugs as novel antibacterials. For example, Nasr et al. [42] demonstrated that a pyrazole derivative (der. 30) proved to be more effective against *Pneumocystis vulgaris* and *K. pneumoniae* than sulfisoxazole and gentamycin. Among 4-(4-formyl-3-phenyl-1*H*-pyrazol-1-yl)benzoic acid derivatives, some of the identified compounds showed antibacterial activity against *A. baumanii* with an MIC of 4 µg/mL [28]. Furthermore, according to Gomes et al. [43], of twenty-one freshly synthesized 1,4-naphthoquinones linked to 1,2,3-1*H*-triazoles, four (**9e**, **9h**, **9i** and **9j**) proved to possess antibacterial activity against *S. mutans* from oral cavities with IZs of 18.66–29.00 mm. The results also showed no toxic effects for these compounds, which possibly increases their potential for application in practice. 1,2,4-triazolidine-3-thiones (Figure 6) exerted antibacterial activity against the ESKAPE list of pathogenic bacteria. Furthermore, binaphthyl-1,2,3-triazole peptidomimetics were efficient against *A. baumannii* with an MIC of 4 g/mL. Along with this, cationic biaryl 1,2,3-triazolyl peptidomimetic derivatives moderately inhibited the growth of *A. baumannii* [28]. Antibacterial but not virulence-targeting activity was also demonstrated in a study by Sapijanskaite-Banevic et al. [44]. In order to create substituted 1-phenyl-5-oxopyrrolidine (Figure 6) derivatives with benzimidazole, oxadiazole, triazole, dihydrazone, and dithiosemicarbazide moieties in the structure, p-aminobenzoic acid (Figure 6) was employed. Using different assays, the antimicrobial activity of each drug was assessed in vitro against *S. aureus*, *Bacillus cereus*, *L. monocytogenes*, *Salmonella enteritidis*, *E. coli*, and *P. aeruginosa*. This work demonstrated the potent bactericidal effects of benzimidazoles and derivatives of amino acids, with some of the compounds exceeding the activity of ampicillin. In the field of medicinal chemistry, combining two or more pharmacological groups into a single molecule is a new approach to drug discovery [45]. As demostrated by Dawoud et al. [46], a novel group of heterocyclic compounds merged using a indazolylthiazole moiety was evaluated for their antimicrobial potential. The obtained results showed that four of the compounds exhibited antibacterial effects, with the strongest activity observed against *Streptococcus mutans* and *P. aeruginosa*. Furthermore, these novel compounds showed virulence-targeting activity, with high antibiofilm potential. Srikanth et al. [28] also suggested that aminothiazolyl berberine (Figure 6) affects the activity of the DNA gyrase of MDR *A. baumannii* strains, exerting remarkable activitiy at an MIC of 2 nmol/mL. Among oxazole/benzisoxazole-based compounds, N-(2-(1*H*-imidazol-4-yl)ethyl)-2-(2,3-dihydroxyphenyl)-*N*-hydroxy-5-methyloxazole-4-carboxamide showed antibacterial activity against *A. Baumannii*, with an MIC of 2 µg/mL (strains UNT190 and UNT197) [28].

## 4. Thiophenes

**Figure 7:**
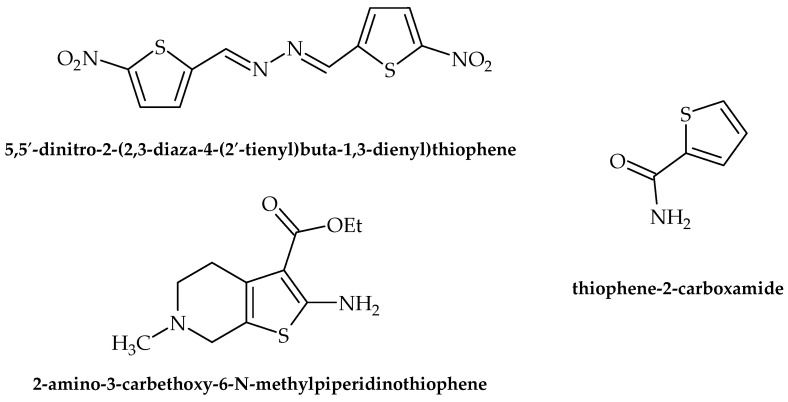
Chemical structures of 5,5′-dinitro-2-(2,3-diaza-4-(2′-tienyl)buta-1,3-dienyl)thiophene, 2-amino-3-carbethoxy-6-*N* methyl piperidino thiophene, and thiophene-2-carboxamide.

Thiophenes and related derivatives are rather versatile heterocyclic compounds with various applications in medicine and drug discovery. With a wide range of bioactive properties, they have been shown to possess remarkable anti-inflammatory, antianxiety, antimicrobial, antioxidant, and other activities. Furthermore, they have long been present on the market as commercial therapeutics, for example, tipeptidine, dorzolamide, and citizolam. However, only data relating to the scope of this review (i.e., antibacterial activity) are presented here. According to Rando et al. [47], 5,5′-dinitro-2-(2,3-diaza-4-(2′-tienyl)buta-1,3-dienyl)thiophene (Figure 7) possesses promising antituberculosis activity, as it inhibited the growth of pathogenic *Mycobacterium avium* and *M. kansasei*. This compound showed notable levels of mutagenicity as well, which limits its potential for application in clinical practice. Moreover, antimicrobial activity against *S. aureus* was observed by Scotti et al. [48], achieved by targeting RNA polymerase.

According to Ramalingam et al. [49], 2-amino-3-carbethoxy-6-*N* methyl piperidino thiophene (Figure 7) was used as a starting point for the synthesis of novel compounds to be tested for their antibacterial potential using the Kirby–Baurer method. Among the tested compounds, twelve were found to be potent against *B. subtilis* and *E. coli*.

The most recent research of Metwally et al. [50] indicated that thiophene-2-carboxamide (Figure 7) derivatives showed antibacterial properties, with *S. aureus*, *B. subtilis*, *E. coli*, and *P. aeruginosa* being the most susceptible to the activity of the tested compounds. While the results showed no indication of targeting virulence factors, the very fact that the novel synthesized compounds possessed microbicidal activity with no targeted virulence factors whatsoever suggests that there is still hope for old-fashioned drugs as antimicrobials.

The types of activity against pathogenic bacteria presented by indoles, azoles, and thiophenes are presented in Table 1.

## 5. Pleuromutilin Derivatives

**Figure 8:**
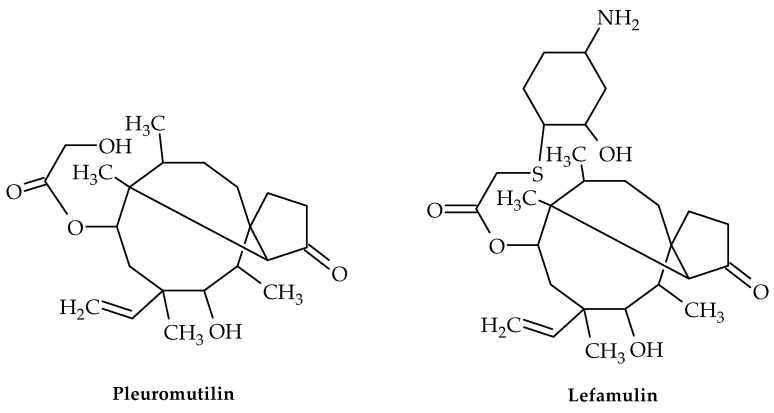
Chemical structures of pleuromutilin and lefamulin.

Pleuromutilin (Figure 8), a diterpenoid secondary metabolite with a tricyclic structure, was initially discovered in *Pleurotus passeckerianus* and *P. mutilis* mushrooms in 1951 [51]. This compound and its derivatives demonstrated strong antibacterial efficacy against Gram-positive bacteria, mycoplasma, and chlamydia [52] by interacting with the peptidyl transferase core (PTC) of bacterial ribosomes and blocking protein synthesis [53,54].

Pleuromutilins bind to the PTC and compete for binding with the 16-atom macrolide and peptidyltransferase inhibitor carbomycin (but not with the 14-atom macrolide erythromycin) [55], inhibiting the formation of peptide bonds [56]. The X-ray crystallography of ribosome-drug complexes was used to identify the precise nature of pleuromutilin binding to the ribosome [57].

Four semi-synthetic derivatives of pleuromutilin have so far received approval for use in the treatment of infectious disorders, including lefamulin (Figure 8) for the treatment of adult community-acquired bacterial pneumonia (CABP) [58], tiamulin and valnemulin for use in veterinary medicine, and retapamulin for use as an antibiotic in the treatment of human skin infections [59,60]. Lefamulin is the only pleuromutilin derivative that has been demonstrated to inhibit the *S. aureus* cfr (chloramphenicol–florfenicol resistance gene) strain [61].

Chemists have worked very hard to create pleuromutilin derivatives due to its unique mechanism of action and promising antibacterial properties [62].

The gene cluster for pleuromutilin has been described and functionally characterized with regard to its production [63]. The creation of new pleuromutilin-based antibiotics will be aided by the identification of new pleuromutilin derivatives [64].

Lefamulin interferes with the peptidyl transferase center of the 50S ribosome by specifically binding at the A- and P-sites, blocking the formation of peptide bonds. This interferes with the production of bacterial proteins [54].

Lefamulin uses a special induced fit mechanism to close the binding pocket within the ribosome, ensuring the tight binding of the drug to the target site, even though this mechanism of action is similar to that of the oxazolidinones and can actually compete with the phenicols for the same binding site [54]. This is a unique strategy for preventing bacterial peptide chain elongation, especially with the creation of the first peptide bond; however, lefamulin is ineffective once elongation has begun [65]. With the exception of *M. pneumoniae*, lefamulin presents bacteriostatic characteristics against the majority of species [66]. Lefamulin has exhibited action against all aerobic Gram-positive organisms, except *E. faecalis* [54].

Likewise, methicillin-resistant *S. aureus* (MRSA), heterogeneous VISA (hVISA), vancomycin-resistant *S. aureus* (VRSA), penicillin-resistant *S. pneumoniae* (PRSP), MDR *S. pneumoniae*, and vancomycin-resistant *E. faecalis* (VRE) are among the resistant Gram-positive organisms against which lefamulin is effective [54,67,68].

**Figure 9:**
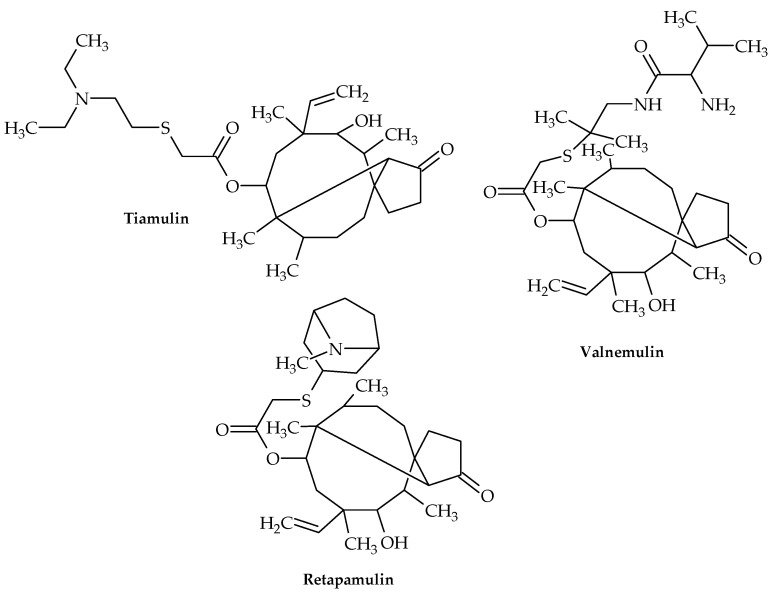
Chemical structures of tiamulin, valnemulin, and retapamulin.

Tiamulin and valnemulin (Figure 9) attach to the bacterial 50S ribosomal subunit to prevent protein synthesis. It has been shown that these medications interact with 23S RNA’s domain V and are potent inhibitors of peptidyl transferase, leaving distinct chemical traces at the nucleotides A2058-9, U2506, and U2584-5. All of these nucleotides are at or near the PTC and have been linked to the binding of several antibiotics. The majority of them are well conserved both phylogenetically and functionally [69].

These two compounds can bind alongside the macrolide erythromycin but compete with the macrolide carbomycin, which is a peptidyl transferase inhibitor, according to competitive footprinting. In order to impede the proper placement of the CCA ends of tRNAs for peptide transfer, these two chemicals interact with the rRNA in the peptidyl transferase slot on the ribosomes. Although ribosomal protein uL3 is located adjacent to the tiamulin binding site without coming into contact with the medication, tiamulin only interacts with rRNA residues [70]. Accordingly, tiamulin binds to the 50s subunit’s A site, and the acetic acid tail extends to the P site, interfering with the formation of peptide bonds [70].

Retapamulin (Figure 9) is a pleuromutilin antibiotic that blocks the formation of the 50S ribosomal unit in bacteria, hence inhibiting the production of proteins [71]. It is effective against Gram-positive pathogens, and since 2007, a topical preparation has been licensed in the US for treating skin and soft tissue infections in adults and children older than 9 months [72].

Retapamulin has demonstrated remarkable in vitro and in vivo action against MRSA and MSSA strains of *S. aureus* in prior studies [73] and has also shown good outcomes against mupirocin-resistant MRSA [72].

## 6. Albocyclin and General Lactone Derivatives

A class of substances known as lactones is commonly present in nature [74]. Chemically, they can be categorized as variously sized intramolecular esters of hydroxycarboxylic acids. The most prevalent are the lactones with five- and six-membered rings due to the stability of the ring structure [75]. However, alternative ring sizes of lactones can also be extracted from natural sources or produced chemically [76].

Lactones are a very fascinating group that demonstrates various significant biological characteristics as a result of its diversity [77].

The main structure of the lactones group has recently been modified to create new analogs with stronger or different responses. These new analogs can exhibit a toxic effect on the cells of pathogenic bacteria and serve as an alternative to the widely used antibiotics [78].

It is known that bacteriostatic properties are exhibited by substances in which the lactone moiety is present in a small ring, e.g., xanthatin [79], a bicyclic lactone isolated from *Xanthium pensylvanicum* and *X. strumarium*, which is active against *S. aureus*, including MRSA-resistant methicillin strains [80].

**Figure 10:**
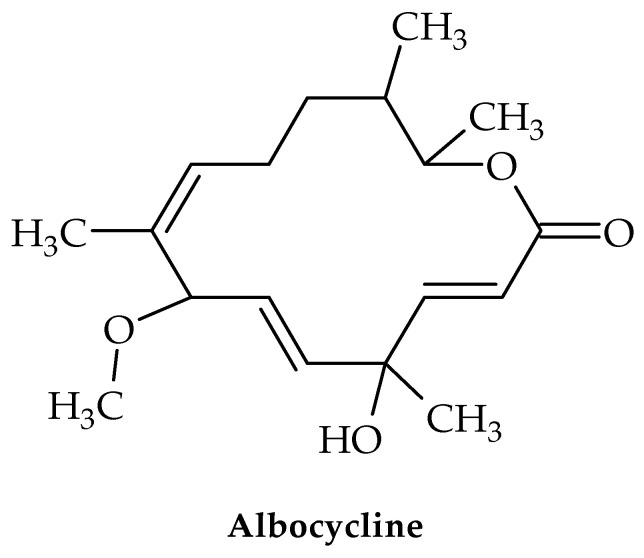
Chemical structure of albocycline.

Several strains of *Streptomyces* produce albocycline—a 14-membered macrolactone (Figure 10) [81]. This compound has shown in vitro antimicrobial activity against MRSA and VRSA equipotent to vancomycin [82,83]. Despite this, albocycline may represent a solution for the treatment of infections caused by *S. aureus* species.

A structural motif in the macrolide family of antibiotics, the 14-membered macrolactone of albocycline indicates that it targets the bacterial ribosome and thereby inhibits translation [84].

Albocycline, however, blocks the incorporation of radiolabeled *N*-acetylglucosamine ([3H]GlcNAc) into the peptidoglycan (PG), the protective polymer surrounding bacterial cells, according to research by Tomoda et al. The first component of bacterial PG production, *N*-acetylglucosamine (UDP-GlcNAc), accumulates as a result of albocycline’s inhibition [82,85].

Due to albocycline’s non-toxicity in mice and humans, in vivo investigations have suggested increased interest in the drug for potential therapeutic uses. Using human HepG2 hepatocellular liver cancer cells, the authors of [85] showed that albocycline was not harmful to human cells at a final concentration of less than 64 g/mL [83].

## 7. Glycopeptides

A class of non-ribosomal cyclic or polycyclic peptides known as glycopeptide antibiotics prevents the formation of Gram-positive bacterial cell walls. These substances function as substrate binders (of cell-wall precursors) as opposed to active-site enzyme inhibitors, unlike other antimicrobial classes [86,87,88].

By attacking lipid II (which represents a peptidoglycan-repeat unit that is related to the lipid transporter), glycopeptide antibiotics prevent Gram-positive bacteria from synthesizing PG. As a result, the lipid transporter shared by peptidoglycan and wall teichoic acid (WTA) biosynthesis, bactoprenol phosphate, cannot be recycled [89]. With each contributing almost 50% of the dry cell-wall weight, PG and WTA are two important parts of the cell wall. Through host attachment, colonization, infection, biofilm development, and the recruitment of penicillin-binding proteins (PBPs) to the septum during cell division, WTA plays a significant role in the pathogenicity of microbes [90]. Consequently, it serves as a desirable target for the creation of new antibiotics [89].

**Figure 11:**
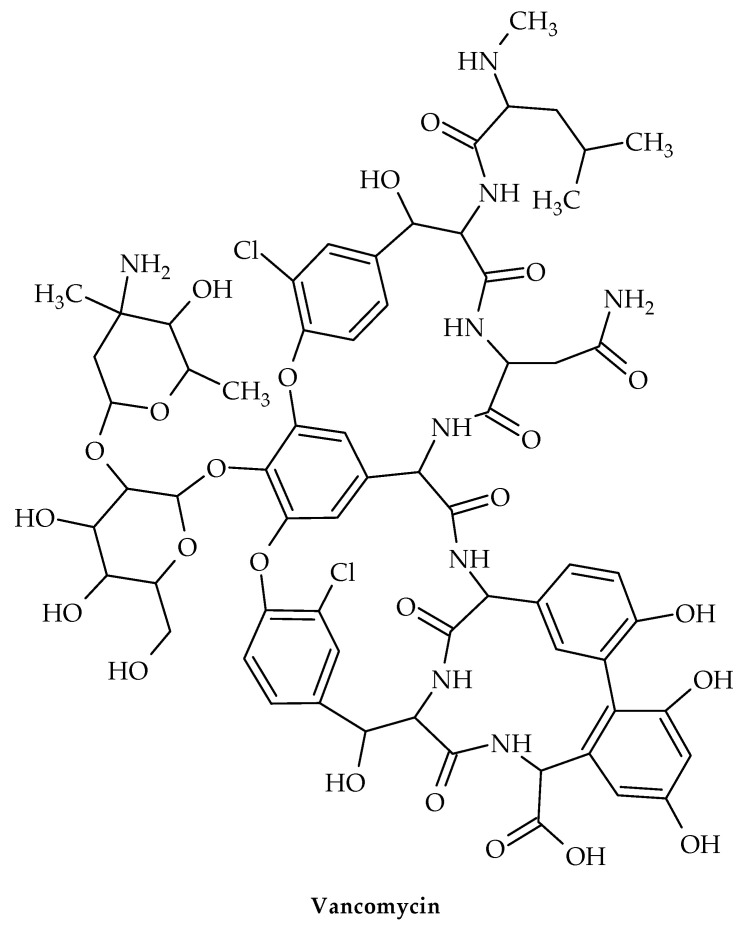
Chemical structure of vancomycin.

**Figure 12:**
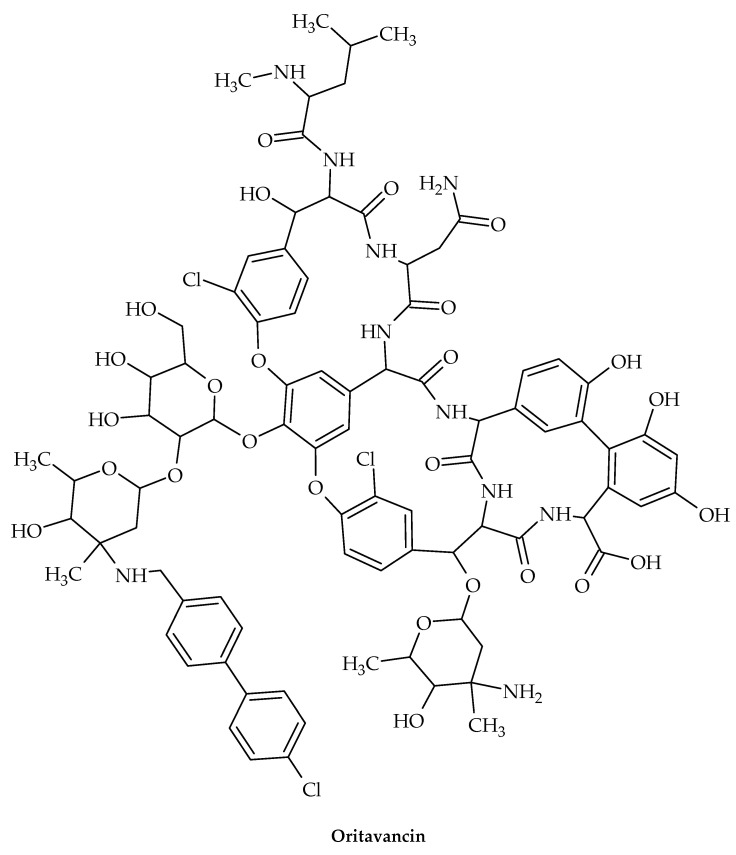
Chemical structure of oritavancin.

**Figure 13:**
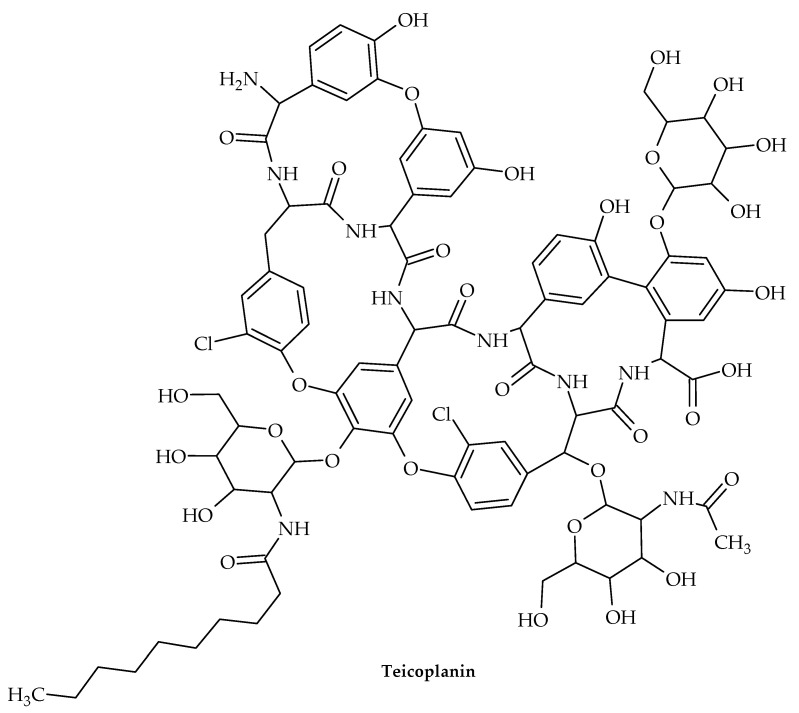
Chemical structure of teicoplanin.

**Figure 14:**
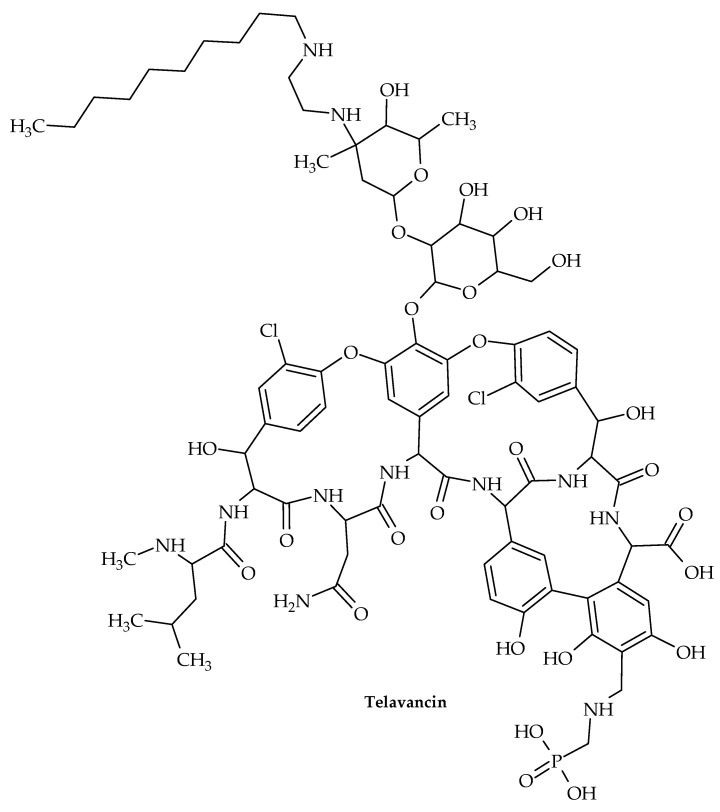
Chemical structure of telavancin.

**Figure 15:**
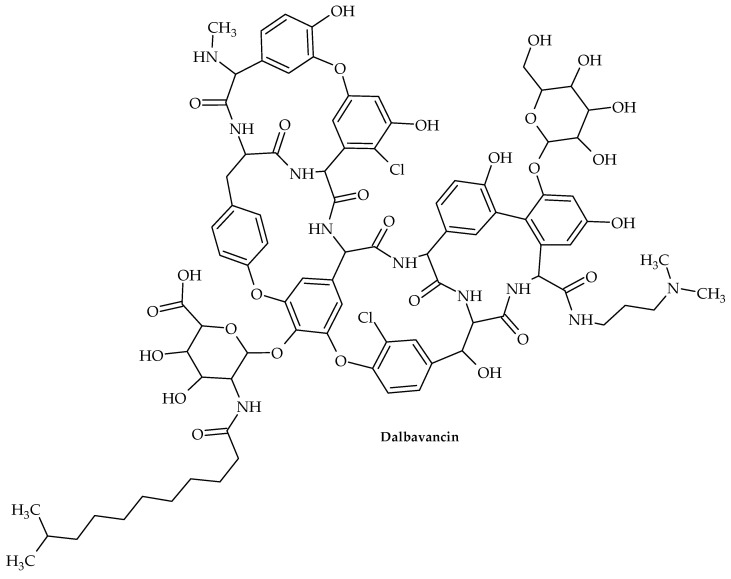
Chemical structure of dalbavancin.

The oldest member of the class is vancomycin (Figure 11), while the more recent lipoglycopeptide derivatives oritavancin, teicoplanin, telavancin, and dalbavancin (Figure 12, Figure 13, Figure 14 and Figure 15) were developed specifically to boost antibacterial activity, sometimes via secondary modes of action.

The transglycosylation stage of PG production, which is necessary to replenish the lipid transporter, is prevented by glycopeptide antibiotic binding to lipid II. Therefore, for instance, when vancomycin is added to *S. aureus* during growth, Park’s nucleotide, a cytoplasmic PG-precursor, accumulates [91]. Vancomycin binding to lipid II is an efficient way to suppress both PG and wall teichoic acid biosynthesis in *S. aureus* [89], since C55 is present in a surprisingly low number of copies per bacterium [92] and is a shared transporter needed in these processes [93].

Vancomycin is used to treat acute infections caused by Gram-positive organisms. By attaching to the D-Ala-D-Ala terminus of lipid II, a PG precursor tethered to the cell membrane by the lipid transporter bactoprenol-phosphate, vancomycin suppresses the formation of PG (C55-P). To stop C55-P regeneration, vancomycin-bound lipid II is sequestered from the PG biosynthesis transglycosylation step. Vancomycin’s sequestration of lipid II causes the cytoplasmic buildup of Park’s nucleotide [91], a cytoplasmic PG precursor, because C55 is present in bacteria in low concentrations [94]. When the dipeptide is swapped out for a depsipeptide D-Ala-D-Lac, vancomycin is unable to attach to the D-Ala-D-Ala terminus of lipid II in VRE [95].

As a result of investigating the structure–activity relationship of chloroeremomycin to combat vancomycin resistance, oritavancin was discovered [96], a semi-synthetic lipoglycopeptide that has potent antimicrobial effects against vancomycin-resistant organisms such as VRE and *S. aureus* resistant to vancomycin (VRSA) [97].

Oritavancin is currently a top therapeutic option for treating serious infections brought on by Gram-positive organisms that are multi-drug-resistant, such MRSA [89]. Oritavancin’s chemical composition differs from vancomycin’s due to the inclusion of a *N*-alkylated chlorobiphenyl side chain in the drug sugar’s epivancosamine. In general, adding a hydrophobic side chain to the glycopeptide disaccharide greatly increases the medications’ overall effectiveness and revives their activity against vancomycin-resistant bacteria. By using solid-state NMR to structurally characterize the binding site of these disaccharide-modified glycopeptides in *S. aureus* [98] and *E. faecium* [99] intact whole cells, it was discovered that the drug’s hydrophobic side chain creates a secondary binding site. The lipoglycopeptides can target the cross-linked PG-bridge structure using this secondary binding site to aid in binding [100]. Oritavancin’s binding to the developing PG prevents transpeptidase from effectively recognizing the PG template, which is necessary for effective PG cross-linking during cell-wall synthesis [101].

Teicoplanin is used to treat multidrug-resistant Gram-positive bacteria, such as MRSA and *Enterococci,* that are responsible for life-threatening infectious illnesses [102]. This glycopeptide antibiotic was initially isolated from *Actinoplanes teichomyceticus*, which was identified in 1978 from an Indian soil sample [103].

Teicoplanin shares structural similarities with vancomycin but differs in that it does not contain a lipid. Both antibiotics work by forming hydrogen bonds with the D-Ala-D-Ala C-terminus of the pentapeptide substrate to prevent the formation of the peptidoglycan chains that make up bacterial cell walls [104]. The hydrophobic lipid chain of this pentapeptide substrate is also known to interact with teicoplanin, placing the antibiotic next to the peptidoglycan [102,105].

Derivatives of teicoplanin have also been shown to form nanoscale aggregates in aqueous solution [106], thereby achieving increased binding power [107].

The oral and topical routes of administration for teicoplanin may result in poor permeability across the epithelial lining due to this concentration-dependent aggregation, and the aggregated form may reduce effective concentrations on certain sites, necessitating a higher dose and ultimately causing bacteria to develop resistance [108].

Another lipoglycopeptide derivative of vancomycin is telavancin (TD-6424). This was developed as a cutting-edge treatment for MRSA and other resistant Gram-positive bacterial infections [109]. The United States Food and Drug Administration (USFDA) granted telavancin approval in 2009 for the treatment of difficult skin and skin structure infections (cSSSIs) caused by Gram-positive bacteria, including MRSA, *S. aureus*, *Streptococcus agalactiae*, *S. pyogenes*, the *S. anginosus* group, and *E. faecalis* [110,111].

Two modes of action for telavancin have been suggested. Telavancin achieves bactericidal activity by interacting with the C-terminal *d*-alanyl-*d*-alanine residue on bacterial cell-wall peptidoglycan precursors, just like vancomycin. This interaction significantly alters the phases of cell-wall formation that include the polymerization of peptidoglycan (transglycosylation) and subsequent cross-linking (transpeptidation) [112]. Telavancin is 10-times more effective than vancomycin at inhibiting peptidoglycan production in intact MRSA cells because it strongly inhibits peptidoglycan generation at the transglycosylase stage.

Furthermore, a second mechanism of action has been mentioned. The depolarization of the bacterial cell membrane is involved, which affects how the cell membrane functions. Given that so few other glycopeptides are thought to function in this way, this dual method of action is of special importance [109]. The interaction of the lipophilic decylaminoethyl moiety of telavancin with the lipid bilayer of the bacterial cell membrane is thought to be the process by which telavancin disrupts cell membranes, albeit this is not fully understood [113]. Telavancin’s affinity for lipid II, a molecule found in bacterial cell membranes, is facilitated by this lipophilic substance.

By disrupting the bacterial cell-wall transglycosylation pathway rather than the bacterial cell-wall transpeptidation mechanism, where vancomycin preferentially binds, telavancin is able to enter the bacterial cell with ease [114].

According to reports, lipid II binding is necessary for telavancin to cause membrane depolarization in *S. aureus*. This might not, however, accurately reflect the crucial phase of bacterial membrane disruption [115]. The loss of potassium ions and cytoplasmic adenosine triphosphate (ATP) may also be related to membrane depolarization. Telavancin’s faster bactericidal impact compared to vancomycin may be caused by this alternative method of action, which only affects bacterial cell membranes and not mammalian cells [112].

Dalbavancin is a semisynthetic derivative of teicoplanin. It is active against most pathogenic Gram-positive organisms, including *Streptococcus* spp., *E. faecalis*, *E. faecium*, MSSA, MRSA, and vancomycin-intermediate *S. aureus*. However, it has poor activity against vancomycin-resistant *S. aureus* and VRE [116].

Similarly to vancomycin and other glycopeptides, dalbavancin inhibits cell-wall formation by interacting with the D-alanyl-D-alanine terminus in the bacterial cell-wall peptidoglycan and blocking cross-linking.

In the USA and Europe, acute bacterial skin and skin structure infections (ABSSSIs) are the only conditions for which dalbavancin is currently licensed [117].

## 8. Chalcones

**Figure 16:**
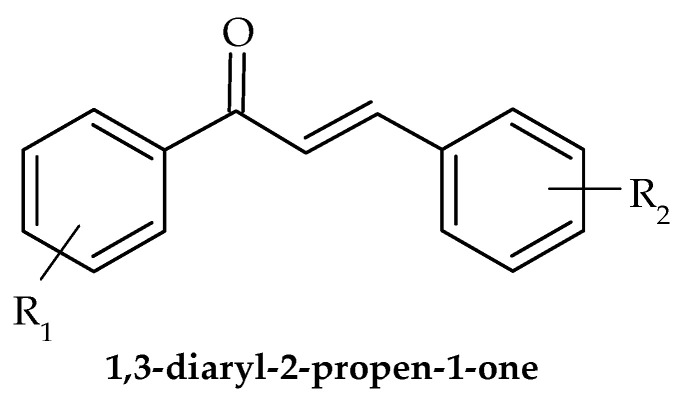
General structure of 1,3-diaryl-2-propen-1-ones.

Due to the hues of the majority of naturally occurring chalcones, the name “chalcone” was derived from the Greek word “chalcos”, which means “bronze”. 1,3-diaryl-2-propen-1-one (Figure 16), also referred to as chalconoid, is a chemical building block shared by all chalcone molecules. The trans isomer is thermodynamically more stable than the cis isomer. Through the use of plants and herbs for the treatment of many diseases, such as cancer, inflammation, and diabetes, chalcones have been applied therapeutically for thousands of years. Several chalcone-based substances have received clinical use authorization.

Chalcones are a class of natural and synthetic compounds that have shown promising antivirulence properties against a variety of pathogenic bacteria. With the rise of antibiotic-resistant strains, there is an urgent need to develop alternative therapies that target virulence factors of bacteria, rather than traditional bactericidal approaches. In recent years, several studies have investigated the antivirulence potential of chalcones in resistant bacterial strains.

Several studies have investigated the activity of chalcones against multidrug-resistant *Pseudomonas aeruginosa* and found that they were able to inhibit the expression of virulence genes involved in quorum sensing, motility, and biofilm formation [118,119].

Furthermore, a study on *Acinetobacter baumannii*, a notorious multi-drug resistant pathogen, showed that chalcones exhibited significant antivirulence activity by modulating gene expression, biofilm formation, and virulence traits [120].

These studies suggested that chalcones have potential as antivirulence agents against resistant bacterial strains by targeting various virulence traits. However, further studies are needed to evaluate the efficacy of chalcones in vivo and their potential as a therapeutic option for antibiotic-resistant infections.

Overall, it can be concluded that, since virulence factors are essential for the infection of the host, techniques employed to prevent this process from initiating and search for novel bioactive compounds with these properties are rather appealing.

Along with the synthetic and hemi-synthetic compounds elaborated in this review article, numerous naturally derived compounds have also demonstrated great potential as efficient virulence-targeting compounds. Dehydroabietic acid showed considerable potential against several pathogenic microorganisms, especially *Pseudomonas syringae* pv. *actinidiae*, *Xanthomonas oryzae* pv. *oryzae*, and *Xanthomonas axonopodis* pv. citri [121]. Furthermore, for some natural compounds, a mode of action has even been proposed. For example, the exposure of *Serratia marcescens* to hordenine (25, 50, and 100 g/mL) reduced the synthesis of acyl-homoserine lactones and prevented the development of biofilms. It also increased the susceptibility of preformed biofilms to commercial antibiotic ciprofloxacin by lowering extracellular polysaccharide production and altering membrane permeability. Additionally, the presence of hordenine downregulated expression and affected genes associated with biofilm and QS [122], which may be explored in other matrices. Additionally, even though some compounds do not exert anti-QS activities per se, they can sometimes be easily modified into compounds that do exert various bioactivities, as was argued by Du et al. [123]. This comprehensive review article offered new research solutions; proposed novel strategies; and compared existing results, leading to new conclusions.

## 9. Future Perspectives

The use of novel synthetic and semisynthetic compounds that target virulence factors as antibacterials presents a promising avenue for combating antibiotic resistance. However, there are several challenges that need to be addressed in order to fully realize the potential of this approach. One of the challenges is the identification of new compounds with antibacterial potency that can target virulence factors. Despite recent progress in this area, many of the compounds that have been investigated are not yet ready for clinical use. The process of discovering, developing, and testing new compounds can be time-consuming and expensive, and there is a need for new screening methods and assays to identify potential candidates more efficiently. Another challenge is the optimization of the efficacy and safety of existing compounds. Many of the compounds that have been identified have shown promising results in vitro, but their efficacy in vivo and safety in humans need to be further evaluated. In addition, the development of resistance to these compounds is a potential concern, and efforts must be made to prevent or delay the emergence of resistance. Furthermore, there is a need for an improved understanding of the mechanisms of action of these compounds. Many of the compounds that target virulence factors have complex modes of action that are not yet fully understood. A deeper understanding of these mechanisms could lead to the development of more effective compounds, as well as the identification of new targets for antibacterial therapy.

There are ongoing clinical trials on novel antivirulence drugs that are trying to take the next steps forward within this area, evaluating the safety and efficacy of novel synthetic and semisynthetic compounds. These trials are being conducted by pharmaceutical companies, academic institutions, and government agencies around the world.

Despite the many challenges, the potential benefits of targeting virulence factors as a strategy to combat antibiotic resistance are significant. By reducing the severity of bacterial infections without promoting the development of resistance, this approach could help to extend the lifespan of existing antibiotics and reduce the need for new ones. In addition, the use of antibacterials that target virulence factors could help to reduce the burden of antibiotic-resistant infections, which are a major public health concern.

## 10. Conclusions

The development of novel antibacterials that target virulence factors is an area of active research aimed at addressing the global challenge of antibiotic resistance. The potential benefits of these compounds lie in their ability to attenuate bacterial pathogenesis without necessarily killing the bacteria, thus reducing selective pressure for resistance development. While the field is still in its early stages, the progress made so far is promising. The use of synthetic and semisynthetic compounds has emerged as an important strategy to combat antibiotic resistance. The compounds reviewed in this paper—chalcones, azoles, indoles, thiophenes, terpenoids, glycopeptides, pleuromutilin derivatives, and lactone derivatives—have shown potential as antibacterials mostly targeting virulence traits in resistant strains. Clinical trials evaluating the safety and efficacy of these compounds are ongoing, and their results will provide critical insights into the role of virulence-targeted antibacterials in the management of bacterial infections. However, given the complexity of bacterial pathogenesis and the evolution of resistance mechanisms, the development of novel antibacterials remains a challenging task. Further research is required to identify novel targets and to optimize the efficacy and safety of these compounds. Additionally, efforts are needed to overcome the regulatory and economic hurdles that often hinder the development and commercialization of novel antibacterial agents.

The development of novel antibacterials that target virulence factors offers a promising avenue for combating antibiotic resistance. While there is still much work to be carried out, the progress made so far suggests that these compounds have the potential to play an important role in the management of bacterial infections in the future.

## Figures and Tables

**Table 1:** Selected compounds and their type of activity against pathogenic bacteria.

Group of Compounds	Compound	Bacteria	Type of Activity	Reference
Indole	5-halo-*1H*-indole-2-carboxylic acids	*Listeria monocytogenes*	Inhibits the growth of bacteria	[25]
Indole	indol-2-one with morpholinosulfonyl	*Staphylococcus* *aureus*	Inhibitor of DNA gyrase	[26]
Indole	thiazolo-indolin-2-one	*S. aureus* (ATCC 29213) *P. aeruginosa* (ATCC 9027)	Inhibits biofilm formation	[27]
Indole	d-pyrimido[4,5-b] indole	*Acinetobacter baumanii*	Inhibits the growth of bacteria	[28]
Indole	3-amino indoles	Multi-drug resistant *A. baumanii*	Inhibits the growth of bacteria	[28]
Indole	4-hydroxy-2-pyridone derivatives containing indolyl	Multi-drug resistant *A. baumanii*	Inhibits the growth of bacteria	[28]
Indole	2-hydrazino2-imidazoline	Multi-drug resistant *A. baumanii*	Inhibits the growth of bacteria	[28]
Indole	bis-indolyl methane	Multi-drug resistant *A. baumanii*	Inhibits the growth of bacteria	[28]
Indole	5-iodoindole	*A. baumanii*, *Escherichia coli*, *S. aureus*	Inhibits the growth of bacteria, decreases motility, disrupts biofilm formation	[29]
Indole	3,3′-diindolylmethane	*Cutibacterium acnes* *S. aureus*	Inhibits the growth of bacteria	[30]
Indole	indole terpenoid compound rhodethrin	*Enterococcus faecalis*	Inhibits biofilm formation	[31]
Indole	indole extract from the supernatant of the rhizobacterium *Enterobacter* sp. Zch127	*Proteus mirabilis*	Inhibits biofilm formation	[32]
Indole	4-chloroindole, 5-chloroindole, 5-chloro 2-methyl indole	*E. coli*	Decreases bacterial motility, disrupts biofilm formation	[33]
Indole	4-chloroindole, 6-iodoindole, 5-chloro-2-methyl indole	*Agrobacterium tumefaciens*	Decreases swimming motility, the production of exopolysaccharide and exoprotease, and cell surface hydrophobicity and biofilm formation	[35]
Indole	indole	*V. tasmaniensis* LGP32 and *V. crassostreae* J2-9	Decreases swimming motility, inhibits biofilm formation	[36]
Indole	4-chloroindole, 7-chloroindole, 4-iodoindole, and 7-iodoindole	*V. parahaemolyticus*	Inhibits biofilm formation	[37]
Azole	naphthalimide-containing nitroimidazoles	*A. baumannii*	Inhibits the growth of bacteria	[28]
Azole	itraconazole and fluconazole	*A. baumannii*	Inhibits biofilm formation	[40]
Azole	clotrimazole, econazole	*Streptococcus mutans*	Inhibits biofilm formation	[41]
Azole	pyrazole 30	*Pneumocystis vulgaris Klebsiella pneumoniae*	Inhibits the growth of bacteria	[42]
Azole	1,4-naphthoquinones linked to 1,2,3-*1H*-triazoles—compounds (9e, 9h, 9i, and 9j)	*S. mutans*	Inhibits the growth of bacteria	[43]
Azole	binaphthyl-1,2,3-triazole peptidomimetics	*A. baumannii*	Inhibits the growth of bacteria	[28]
Azole	heterocycle compounds with indazolylthiazole moiety (compounds 2, 3, 7, and 8)	*S. mutans*, *P.aeruginosa*	Inhibits biofilm production	[46]
Azole	*N*-(2-(*1H*-imidazol-4-yl)ethyl)-2-(2,3-dihydroxyphenyl)-*N*-hydroxy-5-methyloxazole-4-carboxamide	*A. baumannii*	Inhibits the growth of bacteria	[28]
Thiophene	5,5′-dinitro-2-(2,3-diaza-4-(2′-tienyl)buta-1,3-dienyl)thiophene	*Mycobacterium avium* *M. kansasei*	Inhibits the growth of bacteria	[47]
Thiophene	2 -amino-3-carbethoxy-6-*N* methyl piperidino thiophene	*B. subtilis* *E. coli*	Inhibits the growth of bacteria	[49]
Thiophene	thiophene-2-carboxamide	*S. aureus*, *B. subtilis*, *E. coli*, *P. aeruginosa*	Inhibits the growth of bacteria	[50]

## Data Availability

Not applicable.
